# Sleep quality of nurses who worked in coping with COVID-19: an integrative review

**DOI:** 10.1590/0034-7167-2023-0007

**Published:** 2023-12-04

**Authors:** Ítalo Arão Pereira Ribeiro, Ana Lívia Castelo Branco de Oliveira, Carla Danielle Araújo Feitosa, Sandra Cristina Pillon, Maria Helena Palucci Marziale, Márcia Astrês Fernandes

**Affiliations:** I Universidade Federal do Piauí. Teresina, Piauí, Brazil; II Universidade de São Paulo. Ribeirão Preto, São Paulo, Brazil

**Keywords:** Nursing, Sleep, COVID-19, Sleep Quality, Occupational Health, Enfermería, Sueño, COVID-19, Calidad del Sueño, Salud Laboral, Enfermagem, Sono, COVID-19, Qualidade do Sono, Saúde do Trabalhador

## Abstract

**Objective:**

to analyze sleep quality of nurses who worked coping with COVID-19 in scientific evidence.

**Methods:**

an integrative review, carried out in seven databases, including studies between December 2021 and June 2022, without language restrictions. The sample consisted of 15 primary studies.

**Results:**

nurses working in hospital, intensive care, outpatient care and teaching institutions constitute a vulnerable group for sleep disorders: latency, duration, efficiency and quality. The disorders identified involved insomnia at varying levels of severity: daytime dysfunction and morning sleepiness. Night work and low capacity for self-care were determinants of impaired sleep patterns.

**Final considerations:**

the COVID-19 pandemic contributed to greater vulnerability of nurses to changes in sleep, requiring strategies for risk management and well-being promotion.

## RESUMO

**Objetivo:**

analisar nas evidências científicas a qualidade do sono de enfermeiros que atuaram no enfrentamento da COVID-19.

**Métodos:**

revisão integrativa, realizada em sete bases de dados, incluindo estudos entre dezembro de 2021 e junho de 2022, sem restrições de idioma. A amostra foi constituída por 15 estudos primários.

**Resultados:**

os enfermeiros atuantes na assistência hospitalar, intensiva, ambulatorial e de instituição de ensino constituem grupo vulnerável para alterações no sono: latência, duração, eficiência e qualidade. Os transtornos identificados envolveram a insônia em níveis variados de gravidade: a disfunção diurna e a sonolência matinal. O trabalho noturno e a baixa capacidade para autocuidado foram determinantes do comprometimento no padrão de sono.

**Considerações finais:**

a pandemia de COVID-19 contribuiu para maior vulnerabilidade do enfermeiro às alterações no sono, exigindo estratégias para gerenciamento do risco e da promoção do bem-estar.

## RESUMEN

**Objetivo:**

analizar la calidad del sueño de enfermeros que actuaron frente a la COVID-19 en evidencia científica.

**Métodos:**

revisión integradora, realizada en siete bases de datos, incluyendo estudios entre diciembre de 2021 y junio de 2022, sin restricciones de idioma. La muestra estuvo compuesta por 15 estudios primarios.

**Resultados:**

los enfermeros que trabajan en instituciones hospitalarias, de cuidados intensivos, de atención ambulatoria y de enseñanza constituyen un grupo vulnerable para los trastornos del sueño: latencia, duración, eficiencia y calidad. Los trastornos identificados involucraron insomnio en diferentes niveles de severidad: disfunción diurna y somnolencia matutina. El trabajo nocturno y la baja capacidad de autocuidado fueron determinantes de los patrones de sueño alterados.

**Consideraciones finales:**

la pandemia de COVID-19 contribuyó para mayor vulnerabilidad de los enfermeros a los cambios en el sueño, requiriendo estrategias de gestión de riesgos y promoción del bienestar.

## INTRODUCTION

Despite scientific advances and the structuring of measures for epidemiological control, the COVID-19 pandemic constituted a global challenge, as it disrupted the social, economic, political, cultural and health contexts as well as having a severe impact on the population’s quality of life and mental health^(1)^.

Described as a predominantly human infection, this disease was first identified in Wuhan, China, becoming a problem of great magnitude as it was recognized by the World Health Organization (WHO) as an international emergency and required the restructuring of care in different contexts and levels of attention^(2-3)^.

Considering the epidemiological impacts that make Brazil the third country in number of confirmed cases and the second in deaths, health professionals’ performance in coping with the pandemic scenario can show, in addition to greater vulnerability to psychological distress, anxiety, depression and stress, severe impairments in sleep pattern and quality^(4)^.

Sleep is a fundamental need for body relaxation, and serves as a favorable stage for repairing the body’s physiological activities. It is a necessary process for the clearance of toxins that can determine the synthesis and modulation of the immune, cardiovascular, reproductive and endocrine response^(5)^. Still, when restorative, it represents an important indicator of quality of life, since it promotes the maintenance of cognitive functions, the preservation of memory and the balance of the systemic functions of the human organism^(6-8)^.

Alteration in its quality and duration among health professionals, especially in the nursing team, is reported in the national and international scenarios. Evidence shows that this phenomenon can be considered a debilitating condition, resulting in reduced functional capacity, absenteeism, loss of productivity, work leave, reduced quality of life and increased risk of suicide^(4,9)^.

The nursing team, as a prevalent professional category in health institutions, is more exposed to extrinsic and intrinsic factors and those related to work dynamics. Significant efforts have been made to describe, study and understand the impacts of its actions in coping with COVID-19. Although the current scenario indicates epidemiological control, understanding sleep quality in this population is fundamental for the development, direction and effectiveness of care strategies and public policies favorable to occupational safety and risk reduction in the work environment.

## OBJECTIVE

To analyze sleep quality of nurses who worked coping with COVID-19 in scientific evidence.

## METHODS

### Ethical aspects

The study dispenses with ethical considerations, as it involves secondary data.

### Study design

This is a structured integrative review, in six stages, based on the theoretical framework proposed by Whittemore and Knafl (2005): theme identification and research question elaboration; search, selection and sampling; data extraction; critical assessment of results; evidence analysis and synthesis; and presentation of knowledge^(10)^. The study followed the Preferred Reporting Items for Systematic Reviews and Meta-Analyses (PRISMA) recommendations^(11)^.

### Research question elaboration

Following the steps indicated, the research question elaboration was based on the PICo^(12)^ strategy, an acronym for Problem, Phenomenon of Interest and Context. Considering these assumptions, this review was conducted by the following question: what is the scientific knowledge about sleep quality of nurses who worked coping with COVID-19?

### Search, selection and sampling

The bibliographic survey was carried out in June 2022, through electronic access to the Medical Literature Analysis and Retrieval System Online (MEDLINE) via PubMed^®^, Cumulative Index to Nursing and Allied Health Literature (CINAHL-Ebsco), Scopus and Web of Science^TM^ via the Coordination for the Improvement of Higher Education Personnel (CAPES - *Coordenação de Aperfeiçoamento de Pessoal de Nível Superior*) Journal Portal in the area with Internet Protocol (IP), recognized at the *Universidade Federal do Piauí*. Moreover, the following databases were consulted: Latin American and Caribbean Literature in Health Sciences (LILACS); Nursing Database (BDENF); and Spanish Bibliographic Index in Health Sciences (IBECS) via the Virtual Health Library (VHL).

To operationalize the search process, controlled and uncontrolled descriptors (keywords) were selected, indexed in the vocabularies of Health Sciences Descriptors (DeCS)^(13)^ and Medical Subject Headings (MeSH)^(14)^. To combine terms, the Boolean operators “AND” and “OR” were used. The descriptors used to operationalize the search as well as the strategy generated in CINAHL are shown in Chart 1.

**Chart 1:** Search terms and strategy generated in CINAHL, 2022

Health Sciences Descriptors		
P	DC	*Enfermeiras e Enfermeiros*
	DNC	Nurses; *Enfermeras y Enfermeros; Enfermeira; Enfermeiro; Enfermeiro Registrado*
I	DC	*Sono*
	DNC	*Sleep; Sueño; Hábito de Dormir*
Co	DC	COVID-19
	DNC	COVID-19; COVID19; *Pandemia COVID-19; Pandemia por COVID-19*
**Medical Subject Headings**		
P	DC	Nurses
	DNC	Nurse; Registered Nurse
I	DC	Sleep
	DNC	Sleeping Habits; Sleep Habit
Co	DC	COVID-19; COVID-19 Pandemic
	DNC	COVID 19; SARS-CoV-2 Infection
**Search expression**		
( (MH “Nurses”) OR “nurses” OR “nurse” OR “Registered Nurses” ) AND ( (MH “Sleep”) OR “sleep” OR “Sleeping Habits” ) AND ( (MH “COVID-19”) OR “COVID 19” OR “SARS-CoV-2 Infection” OR (MH “COVID-19 Pandemic”) OR “COVID-19 Pandemic” )		

Primary studies, which prioritized as an outcome the pattern and sleep quality of nurses who worked coping with COVID-19, published between December 2021 and June 2022, without language delimitation, were included. Studies carried out with the general population or other professional categories as well as those involving other research outcomes were excluded.

The identified studies were exported to EndNote Basic Manager, aiming at removing duplicates^(15)^. Subsequently, two reviewers, independently, assessed the titles and abstracts as well as determined the inclusion potential of the studies. All conflicts were managed by a third reviewer with experience in the area and in the research method. It is noteworthy that the sample composition was defined after full text analysis of all references included.

### Data extraction

Data extraction was guided by a validated instrument, prioritizing the following variables of interest: reference (main author, year of publication, journal and country) and methodological aspects (design, sample and level of evidence) as well as the main recommendations, results and conclusions^(16)^.

### Critical assessment of results

The critical assessment of the studies was carried out by classifying the level of evidence (LoE). To this end, the recommendations proposed by the Oxford Center for Evidence-based Medicine were adopted, which considers: 1A - systematic review of randomized controlled clinical trials; 1B - randomized controlled clinical trial with narrow confidence interval; 1C - therapeutic results of the “all-or-nothing” type; 2A - systematic review of cohort studies; 2B - cohort study (including lower quality randomized clinical trial); 2C - observation of therapeutic results or ecological studies; 3A - systematic review of case-control studies; 3B - case-control study; 4A - case reports (including lower quality cohort or case-control); 5A - expert opinion^(17)^.

### Evidence analysis and synthesis

For evidence analysis and synthesis, descriptive data methods were used. Furthermore, a synoptic table was constructed, according to the variables of interest defined in this review, with a view to characterizing the studies included and presenting the knowledge produced.

## RESULTS

**Figure 1:**
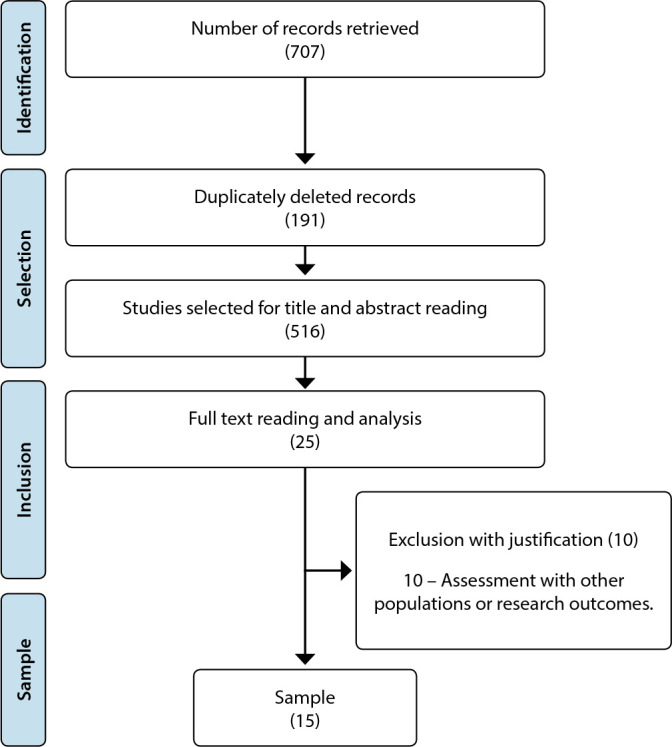
PRISMA flowchart, 2022

The operationalization of the search in the databases of interest favored the identification of 707 records, of which 191 were removed due to duplication. The record was kept in specific health databases, followed by multidisciplinary ones, resulting in the assessment of 516 studies regarding their potential for inclusion. Of these, 25 were selected for reading in full, and 10 met the eligibility criteria, being considered for sample composition. The path of identification, sorting, selection and inclusion is described in Figure 1.

The descriptive analysis of results shows that the epidemiological, individual and collective, care and occupational impacts imposed by the COVID-19 pandemic have a severe impact on nurses’ sleep quality. Accordingly, determinants and associated factors are constant targets of investigations in multiple contexts of the international scenario, such as Turkey and China, which concentrated the largest number of studies.

The studies were published, in their entirety, in English and developed in different care modalities, such as hospital, intensive and outpatient care, concentrating the largest number of investigations and prevalence of nurses with changes in sleep patterns in the hospital area. Still, there was a growing interest of researchers in showing the degree of vulnerability of nurses to sleep disorders, identified by the high number of studies carried out in the last year.

Observational designs, such as the cross-sectional method, predominated. The most common LoE was 2C, which, despite not allowing establishing cause and effect relationships, is relevant for clinical practice and decision-making.

Chart 2 presents the distribution and characterization of the studies included in terms of author, journal of publication, title, year, country in which the study was developed, methodological design adopted and LoE.

**Chart 2:** Characterization of studies included (N=15), 2022

Author and journal	Title	Year	Country	Method/level of evidence
Kandemir D^(18)^ Journal of Clinical Nursing	Analysis of mental health symptoms and insomnia levels of intensive care nurses during the COVID-19 pandemic with a structural equation model	2022	Turkey	Longitudinal 2C
Nazari N^(19)^ BMC Psychiatry	Factors associated with insomnia among frontline nurses during COVID-19: a cross-sectional survey study	2022	Iran	Cross-sectional 2C
Huang L^(20)^ Int J Ment Health Addict	Nurses’ Sleep Quality of “Fangcang” Hospital in China during the COVID-19 Pandemic	2022	China	Cross-sectional 2C
Silva AF^(21)^ Rev. Latino-Am. Enfermagem	Sleep quality, personal and work variables and life habits of hospital nurses	2022	Brazil	Cross-sectional 2C
Sayilan AA^(22)^ Perspect Psychiatr Care	Burnout levels and sleep quality of COVID-19 heroes	2021	Turkey	Cross-sectional 2C
Bernburg M^(23)^ Int J Environ Res Public Health	Stress Perception, Sleep Quality and Work Engagement of German Outpatient Nurses during the COVID-19 Pandemic	2021	Germany	Cross-sectional 2C
Bilgiç S^(24)^ Work	Stress level and sleep quality of nurses during the COVID-19 pandemic	2021	Turkey	Cross-sectional 2C
Sis CA^(25)^ Psychiatr Danub	Comparison of the Fear Levels and Sleep Problems of Nurses Working in Wards Where Patients with and without COVID-19 Are Hospitalized: A Study from Turkey	2021	Turkey	Cross-sectional 2C
Chen X^(26)^ Psychol Res Behav Manag	Sleep Quality and the Depression-Anxiety-Stress State of Frontline Nurses Who Perform Nucleic Acid Sample Collection During COVID-19: A Cross-Sectional Study	2021	China	Cross-sectional 2C
Kim-Godwin YS^(27)^ Res Public Health	Factors influencing sleep quality among female staff nurses during the early COVID-19 pandemic in the United States	2021	USA	Cross-sectional 2C
Li Y.^(28)^ Front Public Health	Predictors of Shift Work Sleep Disorder Among Nurses During the COVID-19 Pandemic: A Multicenter Cross-Sectional Study	2021	China	Cross-sectional 2C
Liang M ^(29)^ Zhong Nan Da Xue	Status quo and influencing factors for anxiety, depression, and insomnia among 4237 nurses in Hunan Province	2021	China	Cross-sectional 2C
Simonetti V^(30)^ J Clin Nurs	Anxiety, sleep disorders and self-efficacy among nurses during COVID-19 pandemic: A large cross-sectional study	2021	Italy	Cross-sectional 2C
Tu ZH^(31)^ Medicine	Sleep quality and mood symptoms in conscripted frontline nurse in Wuhan, China during COVID-19 outbreak: A cross-sectional study	2020	China	Cross-sectional 2C
Zhan Y^(32)^ J Nurs Manag	Factors associated with insomnia among Chinese front-line nurses fighting against COVID-19 in Wuhan: A cross-sectional survey	2020	China	Cross-sectional 2C

*Source: Virtual Health Library, CAPES Journal Portal, 2022.*

Considering the study scenarios, the hospital environment was mentioned in almost its entirety. However, there was one study carried out in an outpatient clinic and another in a teaching institution. Among the identified disorders, alterations in sleep latency, duration and efficiency predominated, contributing to a significant reduction in its quality. Also highlighted were insomnia at varying levels as well as daytime dysfunction and morning sleepiness. The use and dependence on hypnotic drugs to maintain sleep quality was a significant strategy among professionals in the studies that made up the review sample.

Manifestations of poor sleep quality in the studied professionals were permeated by associated factors, such as fear, anxiety, depression, fatigue and stress. These, when associated, determine and intensify the impairment and state of severity in sleep pattern alteration. Predictors for greater sleep impairment involved the direct role of nurses in managing confirmed cases of COVID-19 infection. In addition to this, gender differences, work aspects, social support network and the presence of clinical and psychopathological comorbidities are highlighted. Night work, increase in service indicators, lack of training and low individual capacity for self-regulation and self-care also had a great impact.

Faced with these events, the studies pointed out recommendations to minimize the degree of suffering experienced by nurses working in the pandemic scenario, which mostly included the structuring, assessment and implementation of therapeutic programs favorable to the recognition and management of risk as well as family support network strengthening and well-being promotion (Chart 3).

**Chart 3:** Synthesis of evidence on sleep quality of nurses who worked during the COVID-19 pandemic (N=15), 2022

Reference	Sample (n) and context	Disorders identified	Associated factors	Recommendations
Kandemir D^(18)^	n = 194 Intensive Care Unit	Mild (40.7%), moderate (21.6%) and severe (18.1%) insomnia.	Average weekly working time (49.78 hours), health problem (70.1%), perception that measures to prevent infection are insufficient (78.4%) and depression.	Expansion of the scientific base to propose coping strategies.
Nazari N^(19)^	n = 680 Hospital care	Moderate and severe insomnia (35.8%).	Being female, neuroticism, difficulties in emotional regulation, psychopathological vulnerability.	Therapeutic programs.
Huang L^(20)^	n = 966 Hospital care	Moderate sleep quality.	High demand for work, low support and family support, lack of training and qualification.	Strengthening and building support systems.
Silva AF^(21)^	n = 42 Hospital care	Poor sleep quality (64.3%).	Age group 30-39 years old and living with a partner.	Monitoring strategies.
Sayilan AA^(22)^	n = 384 Hospital care	Insomnia.	Emotional exhaustion, depersonalization, work unit.	Improvements in working conditions and scheduling to ensure adequate sleep.
Bernburg M^(23)^	n = 199 Outpatient care	Significant reduction and impairment of sleep quality.	Stress.	Behavioral, organizational and occupational intervention studies.
Bilgiç S^(24)^	n = 316 Hospital care	Low sleep quality.	Work shift, high stress level, co-worker with COVID-19 and having older family members.	Proactive approaches to psychological support and well-being promotion.
Sis CA^(25)^	n = 211 Hospital care	Low sleep quality.	Fear of acting on the front line and providing care to patients diagnosed with COVID-19.	Individual and institutional support, and better working conditions.
Chen X^(26)^	n = 248 Hospital care	Low latency, efficiency and sleep quality.	Anxiety (81.36%), depression (45.76%) and stress (42.59%).	Crisis management interventions and mental health adjustment.
Kim-Godwin YS^(27)^	n = 215 Teaching and health institution	Daytime dysfunction and dependence on hypnotic drugs.	Full-time job, low perceived health status, and impairment in self-regulation and self-care.	Intervention programs to improve the work environment, health status and work performance.
Li Y^(28)^	n = 4,275 Hospital care	Low sleep quality (48.5%).	Physical fatigue, psychological stress, night work, weekly workload of more than 40 hours, irregular meals and high-intensity physical activity.	Expansion of professional staff and rest hours, promotion of family and social support.
Liang M^(29)^	n = 4,237 Hospital care	Moderate to severe insomnia (12.3%).	Being female, night shift work, low monthly income, and generalized anxiety.	Management policies, psychological support and protection of human resources.
Simonetti V^(30)^	n = 1,005 Hospital care	Moderate sleep quality (71.4%).	Being female and anxiety.	Strategies for recognizing the degree of exposure and risk.
Tu ZH^(31)^	n = 100 Hospital care	Low latency, difficulty initiating or maintaining sleep. Use of hypnotics.	Depression and anxiety.	Behavioral, psychosocial and individual strategies.
Zhan Y^(32)^	n = 1,794 Hospital care	Insomnia (52.8%).	Female sex, chronic diseases, care of confirmed cases, night shifts, family members with severe infection, fear, fatigue and perceived stress.	Interventions based on sleep predictors and determinants.

## DISCUSSION

The present review showed that the epidemiological, care and occupational impacts caused by the COVID-19 pandemic were frequent stressors in the nurses’ work environment, contributing to greater vulnerability to sleep disorders.

The experience of working on the front lines of COVID-19 has been more studied in hospital environments and impacts on health workers’ quality of life are perceived in different areas, including sleep patterns^(18-22,24-26,28-32)^. This brings up the pandemic as a traumatic event, especially in scenarios with more complex care demands, such as intensive care. In these environments, there was a higher rate of anxiety, depression, stress and, therefore, impairments in nurses’ sleep quality. It should also be noted that the activity carried out by health professionals already has important psychological risk factors, which were intensified with the advent of the pandemic^(33)^.

In this regard, it is worth noting that sleep is a restorative tool for the human body and that it promotes health and well-being^(22)^. However, through the losses presented and the conjuncture of related disturbances, the impacts of the results presented on workers’ mental and physical health, which, in the background, affects the quality of care and, therefore, reverberates in axes such as patient safety.

Corroborating the statement, scholars point to the concern with patient safety, which, in addition to being a mission of health professionals, is perceived by patients and can influence their engagement and that of their families regarding safe practices. Patients, in general, perceive especially the limitations arising from work and human relationships in hospitalization scenarios^(34)^.

The interaction between the stressors that influence the disorders was a reality perceived by the participants of the studies that composed the sample. Moreover, disturbances were present, characterized by daytime sleepiness and dependence on hypnotics. In this sense, researchers on the subject have investigated the pattern of chemical dependence and abuse of psychoactive substances within health services, an increasingly frequent practice related to work stress^(28-29)^.

Intense work rhythms and the highly critical demands of patients with COVID-19 have made work environments more tense and demand greater attention from professionals, who remain in a state of alert^(20,24,28-30)^. The ineptitude of qualified human resources to deal with patients with the pathology studied here also led professionals to dedicate themselves above their workloads, which allowed more interference in this worker’s health and sleep^(18,29)^.

Among the strategies, continuing education can bring greater security to the work of professionals. Therefore, due to the lack of this tool, vulnerability to iatrogenesis and stress in the environment increases, which reflects in the results of occupational stress highlighted^(20)^. It should also be noted the rigor of infection prevention measures, as the professionals themselves felt vulnerable to contracting the virus and also the possibility of taking it to family and friends^(18)^. It also impacted the precariousness of personal protective equipment and material resources during the pandemic^(35)^. Some factors intrinsic to individuals were associated with the impaired sleep pattern, such as previous health problems^(18,22)^, difficulties in emotional regulation and psychopathological vulnerability^(19)^, low support and family support,^(20)^ impaired ability of self-regulation and self-care^(28)^.

There is a direct relationship between facing adversities such as COVID-19 and low emotional support. Lack of social support was reinforced by quarantine, stigma and fear of contaminating relatives^(36)^. In addition to the fear of contracting the disease, exposure to the news about COVID-19 affected nurses’ mental health and sleep on the front lines of the pandemic^(37)^. Another relevant point was having an older family member at home, which demonstrated a relationship with the fear of acting in the care of patients with COVID-19, which implied stress and changes in nurses’ sleep pattern^(24,25)^. Being female was another variable identified^(19,29-30,32)^.

Concern for co-workers who contracted COVID-19 influenced psychological distress and, consequently, professionals’ sleep pattern^(24)^. Thus, evidence related to the professional burnout syndrome was identified by researchers on the subject and associated with sleep quality: emotional exhaustion and depersonalization^(22)^.

Therefore, an investigation carried out with nursing professionals in a São Paulo hospital perceived the unease related to team member contamination as common, since the majority of the investigated population was infected by COVID-19. In the study, more than half of the professionals mentioned a lack of some type of Personal Protective Equipment at the institution (50.1%), especially N95/PFF2 or surgical mask, impermeable apron and face shield/goggles^(38)^.

It was a consensus among the studies to list strategies to promote sleep quality and reduce occupational stress, such as therapeutic programs^(19)^, sleep monitoring strategies^(21)^, intervention programs to improve work environments, health status and work performance^(18)^. Shortand long-term perspectives are noted when sleep quality is emphasized within an individual and collective context, in which different determinants are considered, especially that of mental health.

Some points that need strengthening were mentioned, such as expanding the scientific base^(18)^, with investments in behavioral intervention studies^(23)^ and strategies for recognizing the degree of exposure and risk^(30)^. Furthermore, regarding the relevant ideas presented in the studies, there is the strengthening of support systems^(20)^, individual and institutional^(25)^, considering proactive approaches of psychological support and well-being^(24,29)^, and mental health crisis management interventions^(27)^. Suggestions on interventions based on sleep predictors and determinants were raised^(28)^.

However, the central axis focused on occupational aspects, highlighting the need for improvements in working conditions and scheduling to ensure adequate sleep^(22,25)^, expanding the staff and rest hours as well as family and social support promotion^(28)^. However, the need for behavioral, psychosocial and individual strategies is consolidated^(31)^.

When it comes to the reduction of anxiety, depression and stress that were highlighted in this review, there is evidence with proposals for non-pharmacological strategies, such as music therapy^(39)^ and mindfulness strategies. Furthermore, the effectiveness of training aimed at emotional regulation has shown an improvement in sleep patterns^(40)^.

In addition to the highlighted strategies, a study shows that auriculotherapy brought significant results in the subjective sleep quality in Brazilian nursing professionals working on the frontline of care. Such benefits go beyond the improvement of sleep latency and duration, habitual efficiency, reduction of sleep disturbances and use of medication to induce sleep^(41)^.

Finally, considering the importance that sleep quality has in maintaining physical and mental health, it is extremely necessary that future interventions - which focus on relieving stressors at work and managing physical conditions that interfere with sleep - be promoted. Thus, it is essential to propose intervention programs to improve the work environment as well as encourage self-care among nurses. Individually, workers must make an effort to implement self-care practices and strategies regarding sleep hygiene. Together, institutions should provide these workers with methods of maintaining sleep, self-care and management of possible influencing factors for its poor quality. In addition, flexible policies must be adopted that meet all workers’ needs, regardless of sector and shifts^(27)^.

### Study limitations

As limitations presented by this research, it is worth highlighting the inclusion and exclusion criteria established by the researchers, excluding other sources of information and data as well as time frame and number of databases used, which restricted further knowledge on the subject. However, such aspects do not compromise the results presented by this study, since they reveal important findings and that can help in the basis for the development of new research on these professionals’ sleep quality in periods of health crises such as COVID-19.

### Contributions to nursing, health, or public policies

The impact brought to health sciences is considered, since it demonstrates occupational illness from elements that signal quality of life, inside and outside work. Understanding the phenomenon of illness can signal ways of changing behavior and structuring the environment to improve sick professionals’ sleep. Thus, the aim is to reach managers and public policies aimed at health workers with a view to their well-being and quality of life at work.

## FINAL CONSIDERATIONS

The epidemiological, care and occupational impacts imposed by the COVID-19 pandemic contributed to a greater vulnerability of nurses to sleep disorders, which were expressed, in this investigation, by changes in sleep latency, duration and efficiency as well as insomnia at varying levels, daytime dysfunction and morning sleepiness. As for female professionals, working on the front line of care and at night, low capacity for self-regulation and self-care showed higher levels of impairment. Also, symptoms of anxiety, depression, fatigue, fear and stress, increased care indicators and lack of training were associated with the investigated outcome. The studies focus on therapeutic, behavioral, cognitive, individual and collective strategies, which are so necessary for risk management and well-being promotion.

It is expected, with this, to instigate new researchers to explore the theme from different perspectives, considering its richness and upward contributions to workers’ health.
